# The Association between 25-Hydroxyvitamin D Concentration and Telomere Length in the Very-Old: The Newcastle 85+ Study

**DOI:** 10.3390/nu13124341

**Published:** 2021-12-01

**Authors:** Sarah Hakeem, Nuno Mendonça, Terry Aspray, Andrew Kingston, Carmen Martin-Ruiz, Louise Robinson, Tom R. Hill

**Affiliations:** 1 Population Health Sciences Institute, Faculty of Medical Sciences, Newcastle University, Newcastle upon Tyne NE2 4HH, UK; S.H.M.Hakeem2@ncl.ac.uk (S.H.); andrew.kingston@ncl.ac.uk (A.K.); a.l.robinson@ncl.ac.uk (L.R.); 2 Human Nutrition Research Centre, Faculty of Medical Sciences, Newcastle University, Newcastle upon Tyne NE2 4HH, UK; 3 College of Nursing, Umm Al-Quraa University, Makkah P.O. Box 715, Saudi Arabia; 4 EpiDoC Unit, CEDOC, NOVA Medical School, Universidade Nova de Lisboa, 1150-082 Lisbon, Portugal; nuno.mendonca@nms.unl.pt; 5 Comprehensive Health Research Centre (CHRC), NOVA Medical School, Universidade Nova de Lisboa, 1150-082 Lisbon, Portugal; 6 Translational and Clinical Research Institute, Faculty of Medical Sciences, Newcastle University, Newcastle upon Tyne NE2 4HH, UK; Terry.Aspray@ncl.ac.uk; 7 Freeman Hospital, NHS, Newcastle upon Tyne NE7 7DN, UK; 8 Bioscreening Core Facility, Faculty of Medical Sciences, Newcastle University, Newcastle upon Tyne NE4 5 PL, UK; carmen.martin-ruiz@ncl.ac.uk

**Keywords:** ageing, vitamin D, telomere length

## Abstract

(1) Introduction: vitamin D may maintain the telomere length, either directly or via the inflammation effect and/or modulating the rate of cell proliferation. Whilst results from cross-sectional studies investigating the association between 25(OH)D concentration and telomere length have been mixed, there is a dearth of data from prospective studies which have assessed these associations. This study aimed to examine the association between 25(OH)D concentration in plasma and telomere length in blood cells in very-old adults (≥85 years old) at baseline, 18 months and 36 months by controlling for related lifestyle factors. (2) Methodology: our prospective cohort study comprised 775 participants from the Newcastle 85+ Study who had 25(OH)D measurements at baseline. Plasma 25(OH)D was stratified as <25 nmol/L (low), 25–50 nmol/L (moderate) and >50 nmol/L (high). Peripheral blood mononuclear cell telomere length was measured by quantitative real-time polymerase chain reaction at baseline, 18 and 36 months from baseline. (3) Results: a positive significant association was found between 25(OH)D concentration and telomere length amongst very-old participants at baseline (95% CI = 12.0–110.3, B = 61.2 ± 5.0, *p* = 0.015). This association was negative at 18 months (95% CI = −59.9–−7.5, B = −33.7 ± 13.3, *p* = 0.012) but was non-significant at 36 months. (4) Conclusion: Circulating 25(OH)D concentration shows inconsistent relationships with telomere length over time in very-old (85+ year old) adults.

## 1. Introduction

**Figure 1:**
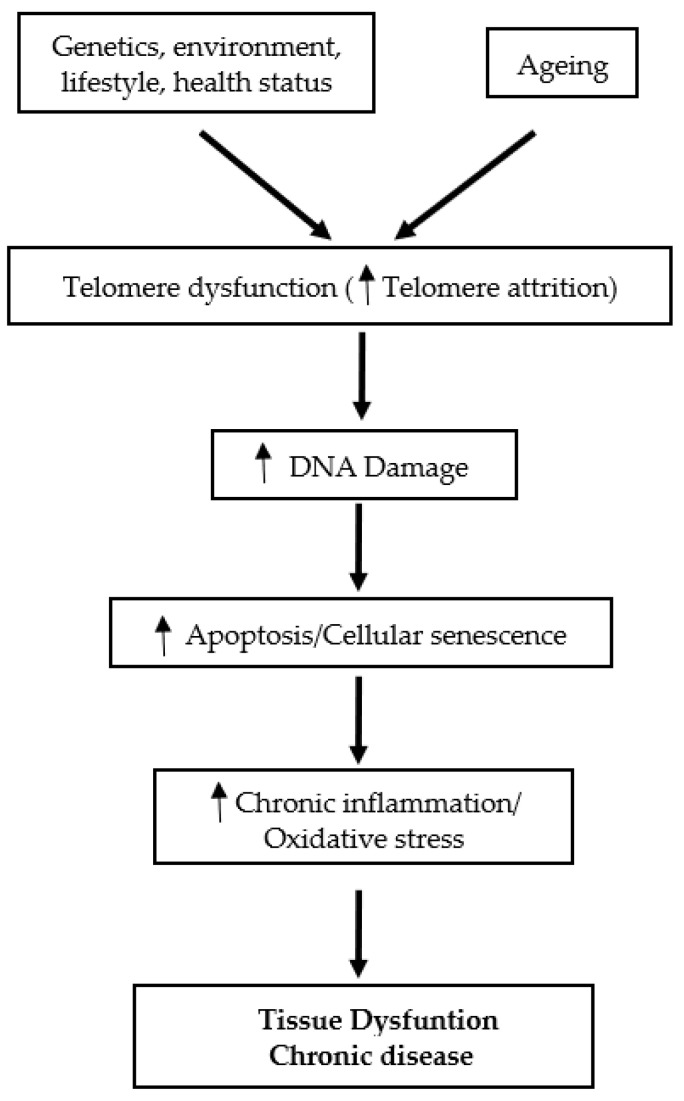
Schematic overview of the role of telomeres in tissue dysfunction and chronic disease.

Telomeres, the specific deoxyribonucleic acid (DNA) protein structures, are the cap at both ends of each chromosome. Telomeres play an essential role in protecting the genome against nucleolytic destruction, recombination, repair, and interchromosomal fusion [1]. Each DNA replication causes telomere shortening and at the point when telomeres reach a critical limit, the cell undergoes senescence and/or apoptosis [2]. The enzyme telomerase plays a key role in the maintenance of telomere length [3]. Age is a well-established factor associated with telomere shortening [3,4]. In humans, telomere length decreases at a rate of 24.8–27.7 base pairs per year [2]. While telomere attrition with age is linked to a variety of age-related diseases and their complications, telomere attrition with ageing has been documented in multiple investigations regardless of the existence of any age-related disorders [3]. These observations support the view that shortened telomeres may serve as a potential candidate biomarker of ageing [3]. Telomere length is also affected by a combination of factors, including sex, genetics and lifestyle [2]. On the other hand, it is purported that the cellular and tissue defects associated with telomere dysfunction are mediated in part by oxidative stress and chronic inflammation mechanisms [5]. Accelerated telomere shortening is associated with mortality and many age-associated diseases, such as cardiovascular diseases, type 2 diabetes mellitus (T2DM), Alzheimer’s disease [1], as well as immune and infectious diseases [6] (Figure 1).

Evidence from animal models as well as human studies have demonstrated that various nutrients (e.g., folate, niacin, vitamin C, magnesium, zinc and omega-3 fatty acids), bioactives (e.g., polyphenols) and whole foods (e.g., tea) may influence telomere length and telomere attrition through mechanisms related to cellular functions including DNA repair and chromosome maintenance, DNA methylation, inflammation, oxidative stress and telomerase activity I (For review see [7]). Vitamin D may influence telomere length through its biologically active hormone 1α,25 dihydroxyvitamin D3 (calcitriol) [8]. Calcitriol is a potent immunosuppressant and has strong anti-inflammatory and anti-proliferative properties mediated in part by its ability to reduce gene expression of inflammatory mediators interleukin- 2 and interferon gamma [7]. The anti-inflammatory and anti-proliferative properties of calcitriol may reduce cell turnover, thus potentially reducing their telomere length attrition [7].

Limited population studies have assessed the association between circulating 25(OH)D concentration [the most commonly used nutritional biomarker of vitamin D] and telomere length [4,8,9,10,11,12,13,14,15,16]. A recent study [9] demonstrated that only at insufficient concentrations of 25(OH)D (50–75 nmol/L), telomerase activity is associated with survival times in CHD patients (mean age 59.9 years old). The findings by Richards et al. [10] and Liu et al. [11] demonstrated a positive association between 25(OH)D concentration and leukocyte telomere length (LTL) in women. On the other hand, a cross-sectional study of 2483 men aged 40–75 years did not observe a positive association between vitamin D biomarkers (25(OH)D and 1,25(OH)2D) and LTL [12]. Liu et al. [13] also found no association between absolute 25(OH)D concentrations and long LTL, for the overall population or the subgroups in the study (men, women, black and white separately) although maintaining a 25(OH)D concentration ≥30 nmol/L was significantly associated with longer LTL in white participants only. Yet, Zhu et al. [14] showed an increase in telomerase activity in 19 overweight African-American women after 16 weeks of vitamin D supplementation.

These conflicting results may be attributed to the differences between gender, race and ages of study participants. For instance, the age range in one study was 48–93 years old (a mean age of 62.8 years old) [13], whereas Richards et al. [10] studied younger women aged 18–79 years old (a mean age of 49.4 years old). Besides, other studies failed to prove the association between 25(OH)D and telomere length in younger participants (mean age 31–39 years old [15] [16]). Generally, these studies included a wide age range of participants with limited number of either very-old adults or participants younger than 70 years old, or participants of only one sex, which limited the generalizability of the findings for those older than 80 years old. Therefore, this study aimed to use the large dataset on both sexes from the Newcastle 85+ Study to examine the association between 25(OH)D concentration and telomere length in very-old adults (>85 years old) [17] at baseline and after 18 and 36 months. We hypothesize that, by controlling related lifestyle factors, a concentration of 25(OH)D <25 nmol/L (used to define vitamin D deficiency) will be associated with shorter telomere length in very-old adults.

## 2. Materials and Methods

### 2.1. Population Sample

The participants were taken from the Newcastle 85+ Study, which included both community-dwelling and institutionalised older adults aged 85 years old at recruitment and living in Newcastle-upon-Tyne and North Tyneside. Commencing in 2006, the study recruited a birth-cohort of more than 1000 adults aged 85 years from North-East England to understand in great detail, the biological, clinical and social determinants of health as the cohort ages. The study seeks to provide extensive data on the relevance of various social, molecular and cellular indicators in contributing to health of very old people [18]. Health assessments, which comprised questionnaires, measurements, function tests and a fasting blood sample as well as general practice (GP) medical records from which to extract data on diagnosed diseases and prescribed medication, were available for the 851 participants. The current paper includes those Newcastle 85+ Study participants (*N* = 775) whose data on health assessment, general practice records and 25(OH)D concentrations were available [18].

### 2.2. Ethical Approval

The study conformed with requirements set in the Declaration of Helsinki. The Newcastle and North Tyneside Research Ethics Committee granted ethical approval, and the participants provided signed informed consent (research project reference number: 06/Q0905/2).

### 2.3. Circulating 25(OH)D Assay and Definition of Vitamin D Status

Following an overnight fast, 40mL of blood was drawn from the participants via the antecubital vein between 7:00 am and 10:30 am. The majority of whole blood samples (95%) were received at the laboratory for processing within 1 h of venepuncture. The total 25(OH)D concentration in serum was estimated by the DiaSorin radio-immunoassay (RIA) kit (DiaSorin Corporation, Stillwater, MN) as described previously [19]. Circulating 25(OH)D measurements were only available at baseline sampling [19].

United Kingdom Scientific Advisory Committee on Nutrition (SACN) cut-offs, which are used for the dietary recommendations for the UK population, are used [20]. A circulating concentration of 25(OH)D < 25 nmol/L was used to indicate the risk of vitamin D deficiency [20].

### 2.4. Telomere Length

Telomere length of peripheral blood mononuclear cell (PBMCs) was measured as an abundance of telomeric template versus a single gene by quantitative real-time PCR. The intra-assay coefficient of variation was 2.7% while the inter-assay coefficient of variation was 5.1% [19]. Four internal control DNA samples were run within each plate to correct for plate–to-plate variation. The measurements were performed in quadruplicate. All PCRs were carried out on an Applied Biosystems 7900HT Fast Real Time PCR machine (Applied Biosystems, Foster City, CA, USA) with a 384-well plate capacity [19].

Aviv et al. 2006 [21] proposes guidance for epidemiological research on the minimum numbers of subjects required for a given age range to determine whether the extrapolated telomere attrition rate of two groups are significantly different. For longitudinal studies assessing telomere length in older adults (>60 years) over time, the sample size required in each test group being compared for 80% power and *p* < 0.05 using a two sided *t*-test is 104 participants [21]. However, given the limited data on telomere attrition in those aged 85–88 years (the age range of the participants in this analysis), any power calculations for very old populations are purely speculative.

### 2.5. HbA1c Measurement

The glycosylated haemoglobin % (HbA1c) was measured using a Tosoh Eurogenetics automated HLC-723G7 HPLC analyser (Tosoh Bioscience, Tokyo, Japan) [19].

### 2.6. Other Health and Lifestyle Variables

#### 2.6.1. Health and Morbidity

Information on health and morbidity was collected from GP medical records by a trained nurse. Diseases were recorded via a predetermined list of key diseases. All diagnoses of listed diseases were scored as present (score 1) or absent (score 0), together with the date of first diagnosis. A simple disease count was used (maximum score 18) from selected chronic diseases (See Supplementary Box S1). The participants were included only if all of the variables were scored as present or absent [18].

#### 2.6.2. Lifestyle

The multidimensional health questionnaire included gender (men or women) and lifestyle factors (smoking, alcohol consumption and physical activity). Using data from the Newcastle 85+ pilot study, a physical activity questionnaire (PAQ) was developed and tested in this age range before being implemented. The participants were divided into three groups: low (scores 0–1), moderate (scores 2–6), and high (scores 7–18) on the PAQ. Physical activity levels are classified based on the frequency and intensity of physical activity performed each week (supplementary data Box S1, available in Age and Ageing online) [22]. For smoking, participants were categorized as non-smokers and occasional and regular smoker. In regard to alcohol consumption, participants were categorized as non-drinkers, moderate drinkers and heavy drinkers. Nutritional status was assessed by calculating body mass index (kg/m^2^) (BMI) from recorded height and weight and included in the models owing to its influence on telomere length [23].

Vitamin D supplement use was divided into two categories: no supplement users and supplement users. Because the only information available on supplement use was the brand and type, micronutrient-containing supplements were assumed to be taken according to the manufacturer’s instructions. The use of vitamin D supplements (yes/no) was collected via the interviewer-administered questionnaire, and prescriptions for vitamin D medicine were retrieved from GP records [24].

### 2.7. Statistical Analysis

The normality of the distributions was assessed by reviewing the histograms and Q-Q plots, and the Shapiro-Wilk test was applied. Normally-distributed, continuous variables are presented as means and standard deviations (SD), while non-Gaussian distributed variables are presented as medians and interquartile ranges (IQR). The categorical data are presented as percentages (with the corresponding sample size). Mann-Whitney and Kruskal-Wallis tests were used for ordered and non-normally distributed continuous variables, and a χ^2^ test for categorical variables.

The concentration of 25(OH)D was not normally distributed (and could not be normalized by transformation, as described previously [25]). In addition, telomere length was not normally distributed (and could not be normalized by transformation either). The 25(OH)D concentration was categorized by SACN cut-offs points for vitamin D [19]: <25 nmol/L (as low). In order to have equal participants distributed between the groups, we used 25–50 nmol/L (as moderate) (used as reference in the analysis) and >50 nmol/L (as high). To examine the association between 25(OH)D and telomere length at baseline, 18 months and 36 months, linear regression was used. The linearity and homoscedasticity assumptions were tested with residual normality versus predicted values plots. Important confounders were selected based on their clinical and theoretical relevance to the telomere length. These variables were then fitted, removed, and refitted until the best possible but parsimonious model was achieved while checking for model fit statistics throughout. Model 1 is an unadjusted model, Model 2 is adjusted for smoking and alcohol consumption, Model 3 is further adjusted for BMI and physical activity, and Model 4 is further adjusted for HbA1c. The models were stratified by sex.

All analyses were performed using IBM’s SPSS Statistics software, version 24 (IBM, New York, NY, USA), and *p* < 0.05 was considered statistically significant.

## 3. Results

### 3.1. Participants’ Characteristics

By using the 3 cut-off of 25(OH)D concentration (low, moderate and high), there are significant differences between men and women, BMI categories, PA level, vitamin D containing medication and supplements usage, their general health rate, telomere length and HbA1c measurement (Table 1). The majority of the participants were women, have normal weight, moderately active, non-alcohol drinker, regular smokers and rate their health as good.

### 3.2. Predictors of Telomere Length

No significant association was found between telomere length and the relative confounders from the literature, such as smoking, alcohol consumption, PA, BMI, vitamin D containing medication usage, supplement, disease count and HbA1c% amongst all the participants. The only significant association was between telomere length and sex (95% CI = 0.000–0.001, *p* < 0.001). In addition, no significant association was found between telomere length and the confounders when the participants were stratified by sex, except for the BMI among the women (95% CI = 0.0034–0.044, *p* = 0.039).

**Table 1:** Participant characteristics by circulating 25(OH)D cut-offs in the Newcastle 85+ Study at baseline.

	Low (*n* = 193)	Moderate (*n* = 302)	High (*n* = 283)	All (*n* = 778)	*p*
Women % (*n*)	64.4 (123)	53.8 (162)	65.7 (186)	60.8 (471)	0.007
BMI					0.015
Underweight % (*n*)	22.2 (39)	30.0 (86)	35.5 (91)	30.0 (216)	
Normal weight % (*n*)	43.8 (77)	46.0 (132)	41.0 (105)	43.7 (314)	
Overweight % (*n*)	19.9 (35)	14.6 (42)	17.2 (44)	16.8 (121)	
Obese % (*n*)	14.2 (25)	14.2 (25)	6.3 (16)	9.5 (68)	
PA					0.001
Low % (*n*)	28.6 (54)	15.4 (46)	24.8 (70)	22.1 (170)	
Moderate % (*n*)	48.1 (91)	131 (44)	38.3 (108)	42.9 (330)	
High % (*n*)	23.3 (44)	40.6 (121)	36.9 (104)	35.0 (269)	
Alcohol drinkers					0.056
Never % (*n*)	42.9 (81)	40.1 (120)	40.7 (113)	41.0 (314)	
Moderate % (*n*)	30.2 (57)	39.5 (118)	41.0 (114)	37.7 (289)	
Heavy % (*n*)	11.6 (22)	10.7 (32)	9.4 (26)	10.4 (80)	
Smoking					0.447
Never % (*n*)	36.6 (70)	33.3 (100)	36.2 (102)	35.2 (272)	
Occasional % (*n*)	4.2 (8)	6.3 (19)	4.6 (13)	5.1 (40)	
Regular % (*n*)	59.2 (113)	60.3 (181)	59.2 (167)	59.6 (461)	
Vitamin D containing medication % (*n*)	0.0 (1)	6 (17)	38 (108)	16.5 (126)	<0.001
Supplement users % (*n*)	4.7 (9)	16.9 (51)	32.5 (92)	19.5 (152)	<0.001
Self-rated health					0.006
Very good % (*n*)	37.7 (72)	40.5 (122)	41.7 (118)	40.3 (312)	
Good % (*n*)	53.9 (103)	56.1 (169)	55.1 (156)	55.2 (428)	
Poor % (*n*)	6.3 (12)	2.1 (6)	2.1 (6)	3.0 (23)	
Disease count mean (SD)	4.9 (1.8)	4.7 (1.6)	4.8 (1.9)	4.8 (1.8)	0.675
Telomere length sample % (*n*)					0.678
At baseline	44.7 (190)	42.3 (291)	42.3 (271)	42.9 (752)	
At 18 months	32.2 (137)	32.6 (224)	33.7 (216)	32.9 (577)	
At 36 months	23.0 (98)	25.0 (172)	23.9 (153)	24.1 (423)	
Telomere length (kb) Median (IQR)					0.006
At baseline	3827.1 (1641)	3721.0 (1094)	4009.5 (1021)	4034.6 (800.1)	
At 18 months	3809.2 (236)	3811.8 (542)	3678.5 (487)	3785.2 (415.5)	
At 36 months	2702.1 (1184)	2718.9 (1142)	2781.3 (842)	2832.7 (741.2)	
HbA1c (%) mean (SD)	6.1 (1.1)	6.0 (0.7)	5.8 (0.6)	5.9 (0.7)	0.025

BMI: body mass index. HbA1c: glycated haemoglobin. Kb: kilo-base pair. *p*: *p*-value. Mann-Whitney U test for continuous non-normally distributed variables or χ2 test for categorical variables. 25(OH)D: <25 nmol/L (low), 25–50 nmol/L (moderate), >50 nmol/L (high).

### 3.3. Circulating 25(OH)D Concentration and Telomere Length among the Very-Old Adults at Baseline

**Figure 2:**
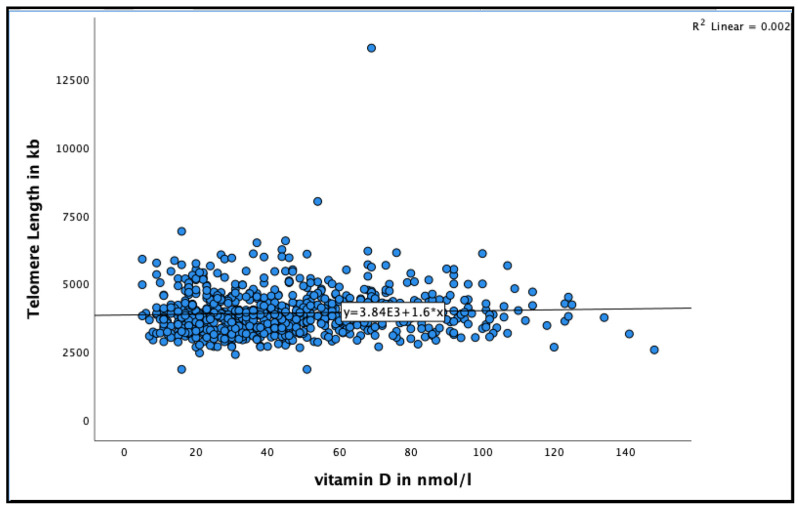
The association between 25(OH)D concentration and telomere length at baseline in the Newcastle 85+ Study.

A positive association was found between 25(OH)D and telomere length in very-old adults (See Figure 2 for the distribution of 25(OH)D concentrations by telomere length at baseline). Participants with 25(OH)D concentration >50 nmol/L had longer telomere length compared to those with concentration <50 nmol/L in the unadjusted model (95% CI = 17.8–109.9, B = 63.9 ± 23.4, *p* = 0.007), and even after adjusting for relevant confounders, such as smoking, alcohol consumption, BMI, physical activity and HbA1C (95% CI = 12.0–110.3, B = 61.29 ± 25.0, *p* = 0.015) (Table 2).

### 3.4. Circulating 25(OH)D Concentration and Telomere Length by Sex

Since sex was the only predictor of telomere length that was found for the current participants, the participants were stratified using this factor. When the participants were stratified by sex (Table 3), the very-old men with concentration between <25 nmol/L were more likely to have shorter telomere length compared to those with concentration 25–50 nmol/L in the unadjusted model (95% CI = 1.9–473.4, B = 237.7 ± 119.7, *p* = 0.048) and even after adjusting for relevant confounders (95% CI = 14.9–521.6, B = 268.3 ± 128.6, *p* = 0.038).

In contrast, the very-old women with 25(OH)D concentration >50 nmol/L were more likely to have longer telomere length compared to those with concentration 25–50 nmol/L in the unadjusted model (95% CI = 15.6–137.3, B = 76.4 ± 30.9, *p* = 0.014). This association continued after further adjustments were made for smoking, alcohol consumption, BMI, and physical activity (95% CI = 6.8–138.1, B = 72.4 ± 33.3, *p* = 0.030) but it disappeared after adjusting for HbA1c (95% CI = −0.7–131.1, B = 65.1 ± 33.5, *p* = 0.053).

### 3.5. Circulating 25(OH)D Concentration and Telomere Length among the Very-Old Adults at 18 Months

**Figure 3:**
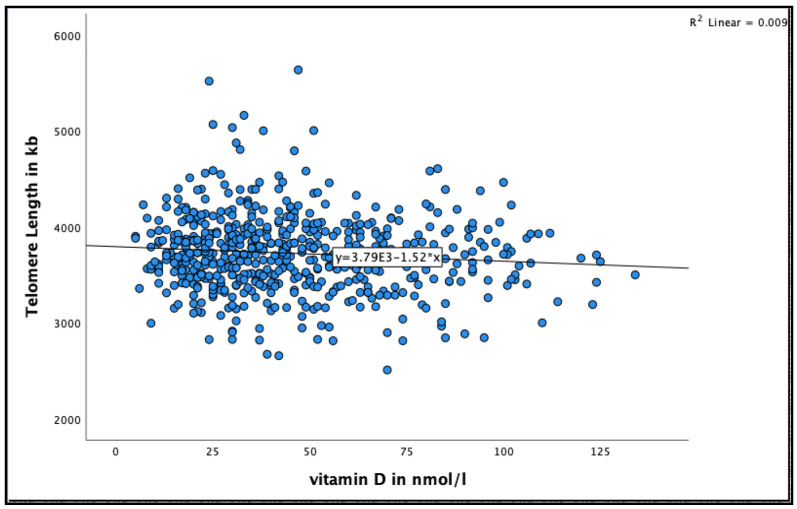
The association between 25(OH)D concentration and telomere length at 18 months in the Newcastle 85+ Study.

A negative significant association was found between 25(OH)D concentration and telomere length at 18 months (See Figure 3 for the distribution of 25(OH)D concentrations by telomere length at 18 months). Very-old participants with 25(OH)D concentration >50 nmol/L were more likely to have shorter telomere length compared to those with concentration 25–50 nmol/L in the unadjusted model (95% CI = −55.9–−5.8, B = −30.9 ± 12.7, *p* = 0.016), and after adjusting for relevant confounders, such as smoking, alcohol consumption, BMI, physical activity and HbA1C HbA1C (95% CI = −59.9–−7.5, B = −33.7 ± 13.3, *p* = 0.012) (Table 4).

### 3.6. Circulating 25(OH)D Concentration and Telomere Length by Sex at 18 Months

When the participants were stratified by sex (Table 5), a negative association was found between 25(OH)D and Telomere Length in the very-old men. Very-old men with concentration >50 nmol/L were more likely to have shorter telomere length compared to those with concentration <50 nmol/L in the unadjusted model (95% CI = −107.3–−25.5, B = −66.2 ± 20.8, *p* = 0.002) and even after adjusting for relevant confounders (95% CI = −107.0–−23.3, B = −65.2 ± 21.2, *p* = 0.002).

However, no significant association was found between 25(OH)D and telomere length in the very-old women at 18 months in both unadjusted and adjusted models (see Table 5).

### 3.7. Circulating 25(OH)D Concentration and Telomere Length among the Very-Old Adults at 36 Months

**Figure 4:**
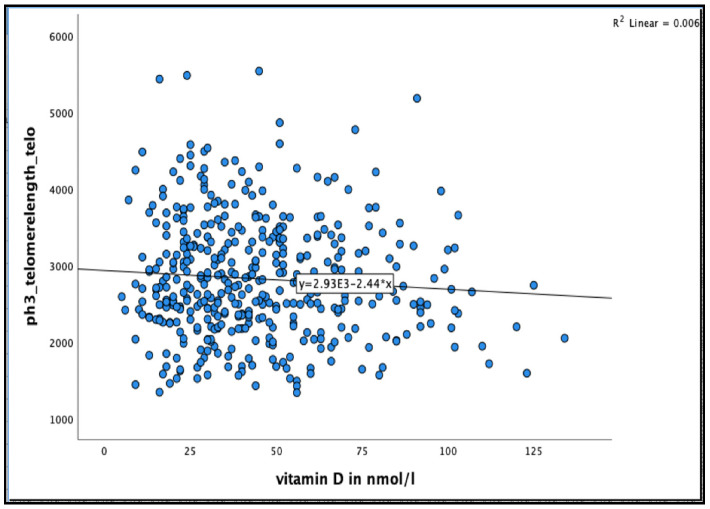
The association between 25(OH)D concentration and Telomere length at 36 months in the Newcastle 85+ Study.

No significant association was found between 25(OH)D concentration and telomere length after 36 months in the unadjusted model, or even after adjusting for relevant confounder smoking, alcohol consumption, BMI, physical activity and HbA1C% in all participants and in men and women separately (see Figure 4 and Table 6 and Table 7)

## 4. Discussion

### 4.1. Main Findings

To our knowledge, this is the first study which has examined the prospective association between circulating 25(OH)D concentration and telomere length in very-old adults (≥85 years). Our results show that in fully adjusted models, whilst there was a significant positive association between 25(OH)D (>50 nmol/L) and telomere length at baseline, the direction of association was reversed after 18 months and absent at 36 months. However, it should be noted that the strength of the positive and negative associations between 25(OH)D concentration and telomere length at baseline and 18, respectively were weak [adjusted R square 0.004 at baseline and 0.020 at 18 months].

### 4.2. Evidence from Other Studies

Telomeres, the specific DNA protein structures, are found at both ends of each chromosome. Their function is to protect the genome from nucleolytic degradation, unnecessary recombination, repair, and interchromosomal fusion [1]. Each DNA replication causes telomere shortening, and when the telomere length reaches a critical limit, the cell undergoes senescence and/or apoptosis [2]. The rate attrition differs between individuals and tissues, and is influenced by multiple factors. Inflammation and oxidative stress are the key determinants of telomere length, and even though some of the factors that heighten oxidative stress and inflammation are genetic, others are environmental in nature, such as smoking, alcohol consumption, obesity and a sedentary lifestyle. Moreover, several dietary factors, such as high energy consumption and the intake of high sugar foods, are also highly associated with inflammation [4,26]. While these lifestyle habits may be difficult to change, vitamin D concentration was easily modifiable through nutritional supplementation or sunlight exposure. Taking all of this into account, we sought to explore and study the association between 25(OH)D concentration and telomere length.

Our findings were in agreement with previous findings from two large studies on women by Richards et al. [10] and Liu et al. [11] ] (*n* = 2160 and *n* = 4604 participants, respectively). Both studies found that a higher 25(OH)D concentration was associated with longer LTL [10,11]. Also, a recent study by Zarei et al. [8] found an interaction between vitamin D and telomerase with regards to their relationship with the survival among 404 CVD patients. On the other hand, the findings from a large community-dwelling study conducted by Liu and colleagues [13], failed to find any association between continuous 25(OH)D concentrations and longer LTL not only for their entire population (*n* = 1154) but also in the white (*n* = 503), black (*n* = 651), female (711), male (447) or race–sex subgroups. However, they found that concentrations of vitamin D ≥30 nmol/L were significantly associated with longer LTL in whites only [13].

In a prospective study of 59 African-American systemic lupus erythematosus patients and their counterpart control subjects shorter telomeres were seen among all subjects with a 25(OH)D concentration <50 nmol/L [16]. Interestingly, the patients who remained vitamin D deficient after three months of follow up (*n* = 29), tended to have shorter telomeres than those patients whose 25-hydroxyvitamin D levels were replete [16], suggestive of a protective role of 25(OH)D in maintaining telomere length. Moreover, a large community-dwelling study conducted by Mazidi and colleagues [7], examined the association between 25(OH)D concentration and telomere length across a broad age range (age: 18–80 years old). The participants were free of any history of diabetes, coronary heart disease, angina, myocardial infarction, stroke or congestive heart failure, in both men (*n* = 2319) and women (*n* = 2668) [7]. A positive association was demonstrated between 25(OH)D concentration and telomere length in the limited-adjusted models. Both studies highlighted the possible role of 25(OH)D concentration in the maintenance of telomere length [7,16].

Our study also demonstrated an association between 25(OH)D and telomere length in men, which is inconsistent with a cross-sectional study in white men (*n* = 2483), which failed to observe an association between any of the vitamin D biomarkers (25(OH)D and 1,25(OH)D) and LTL [12]. However, the participants in the study by Julin et al. [12] were younger than our participants (the mean age was 64.1 years old). Furthermore, they defined 25(OH)D concentration by four quartiles with higher cut-offs (<50 nmol/L was the lowest quartile) while the current study showed that a 25(OH)D concentration >50 nmol/L, was positively associated with telomere length at baseline.

Regarding the contribution of sex to the association between 25(OH)D concentration and telomere length, several biologically plausible explanations for a difference between men and women have been suggested, such as men, in general, having shorter telomeres than women [27]. In addition, estrogen can stimulate the production of telomerase and is a potent antioxidant and regulator of antioxidant genes [28]. Therefore, it should be noted that the differences in sex could contribute toward the association between vitamin D status and telomere length.

Regarding the negative association between 25(OH)D concentration and telomere length at 18 months, the plausible explanations could be that 25(OH)D concentration was only measured at baseline and not at follow up phases. Another explanation could be that concentration >50 nmol/L might not have protective effect on telomere length at very-old age. However, it should be considered that the model is not explaining much of the variation (Adj R2 considered very low). Besides, the 95% CIs were wide even when the relationship was positively significant at baseline indicates a less precise estimate of the relationship. That said, we could not ascertain the protective association of high concentration of 25(OH)D on telomere length in very-old adults in the current population.

There are several potential mechanisms that may explain the association between telomere length and 25(OH)D concentration. Generally, an activated form of vitamin D has autocrine and paracrine roles, including reducing telomere shortening through both anti-inflammatory and antiproliferative mechanisms. First, the active form of vitamin D decreases the mediators of systemic inflammation, such as interleukin-2 and tumor necrosis factor [9]. Furthermore, vitamin D receptor is expressed in the T and B lymphocytes, natural killer cells, and monocytes, which promote the down-regulation of cytokines and other proinflammatory factors. Thus, it follows that vitamin D would attenuate the rate of telomere length attrition [7,29]. In addition, the retinoid x receptor (which is found widely distributed in cells and tissues and acts as the major contributor to vitamin D dependent transcription) may attenuate the relationship between vitamin D and telomere length has other roles in the cell that are independent of the vitamin D pathway. Therefore, the association between one common variant and a long telomere length does not necessarily imply a link between 25(OH)D and telomere length [12].

### 4.3. Strengths and Limitations

The study has several strengths, including its unique design, as well as the fact that the analysis is concentrated on a broadly representative age category of 85 years old; and that the statistical assumptions were met. Another key strength is that the study was adjusted for major potential confounders associated with telomere length (e.g., BMI, physical activity, smoking). It should also be noted however, that the findings reported here should be interpreted with caution due to the following limitations: firstly, its epidemiological design restricts any inference about causal relationships. Secondly, we did not include wider dietary factors as covariates in our models as we had no a priori knowledge from our dataset that these factors could associate with telomere length. As a result, unmeasured or uncontrolled factors may confound the findings, raising the risk of Type I error. Adding more confounders to the fully adjusted model, on the other hand, may have resulted in non-significant (bias) results and decreased power to detect significant relationships. Third, despite having longitudinal telomere length data spanning 36 months, serum 25(OH)D data was only collected at baseline.

## 5. Conclusions

Among the very-old in the Newcastle 85+ cohort study, 25(OH)D concentration was positively associated with telomere length at baseline. However, given the wide 95% CI and the conflicting directions of the associations at 18 months inclined to say that high concentration of 25(OH)D (>50 nmol/L) did not show protective effect on telomere length in very-old adults. In conclusion, high 25(OH)D concentration is positively associated with telomere length but does not have protective effect over time.

## Figures and Tables

**Table 2:** Association between 25(OH)D cut-offs and telomere length at baseline.

Model	25(OH)D	β Coefficient	Adj. R Square	95% CI	*p*
Model 1	Low	84.8	0.007	−67.7, 237.5	0.275
	Moderate	(ref)		(ref)	(ref)
	High	63.9		17.8, 109.9	0.007
Model 2	Low	89.8	0.007	−63.9, 243.6	0.252
	Moderate	(ref)		(ref)	(ref)
	High	67.8		21.6, 114.1	0.004
Model 3	Low	88.5	0.004	−76.0, 253.2	0.291
	Moderate	(ref)		(ref)	(ref)
	High	64.4		15.5, 113.2	0.010
Model 4	Low	77.2	0.004	−88.3, 242.8	0.360
	Moderate	(ref)		(ref)	(ref)
	High	61.2		12.0, 110.3	0.015

CI: confidence interval. *p*: *p*-value. 25(OH)D cut-offs: <25 nmol/L (low), 25–50 nmol/L (moderate) (ref) and >50 nmol/L (high). Ref: reference group. BMI: body mass index. HbA1c: glycated haemoglobin. Model 1 is the unadjusted model. Model 2 is further adjusted for smoking and alcohol. Model 3 is further adjusted for BMI and physical activity. Model 4 is further adjusted for HbA1c%.

**Table 3:** Association between 25(OH)D cut-offs and telomere length by sex at baseline.

Sex	Model	25(OH)D	β Coefficient	Adj. R Square	95% CI	*p*
Men (*n* = 304)	Model 1	Low	237.7	0.009	1.9, 473.4	0.048
		Moderate	(ref)		(ref)	(ref)
		High	56.6		−13.6, 126.9	0.114
	Model 2	Low	267.5	0.007	29.2, 505.7	0.028
		Moderate	(ref)		(ref)	(ref)
		High	63.4		−7.6, 134.6	0.080
	Model 3	Low	262.2	0.004	10.8, 513.5	0.041
		Moderate	(ref)		(ref)	(ref)
		High	68.4		−5.8, 142.7	0.071
	Model 4	Low	268.3	0.004	14.9, 521.6	0.038
		Moderate	(ref)		(ref)	(ref)
		High	71.4		−3.6, 146.4	0.062
Women (*n* = 471)	Model 1	Low	28.8	0.011	−172.1, 229.7	0.778
		Moderate	(ref)		(ref)	(ref)
		High	76.4		15.6, 137.3	0.014
	Model 2	Low	20.8	0.009	−181.2, 223	0.839
		Moderate	(ref)		(ref)	(ref)
		High	79.9		18.8, 141.0	0.010
	Model 3	Low	−5.8	0.010	−225.2, 213.6	0.958
		Moderate	(ref)		(ref)	(ref)
		High	72.4		6.8, 138.1	0.030
	Model 4	Low	−23.3	0.011	−243.0, 196.3	0.835
		Moderate	(ref)		(ref)	(ref)
		High	65.1		−0.7, 131.1	0.053

CI: confidence interval. *p*: *p*-value. 25(OH)D cut-offs: <25 nmol/L (low), 25–50 nmol/L (moderate) (ref) and >50 nmol/L (high). Ref: reference group. BMI: body mass index. HbA1c: glycated haemoglobin. Model 1 is the unadjusted model. Model 2 is further adjusted for smoking and alcohol. Model 3 is further adjusted for BMI and physical activity. Model 4 is further adjusted for HbA1c%.

**Table 4:** Association between 25(OH)D cut-offs and telomere length at 18 months.

Model	25(OH)D	β Coefficient	Adj. R Square	95% CI	*p*
Model 1	Low	−1.1	0.012	−86.3, 84.1	0.979
	Moderate	(ref)		(ref)	(ref)
	High	−30.9		−55.9, −5.8	0.016
Model 2	Low	−2.2	0.022	−87.1, 82.7	0.959
	Moderate	(ref)		(ref)	(ref)
	High	−32.2		−57.2, −7.3	0.011
Model 3	Low	−8.5	0.022	−97.9, 80.8	0.851
	Moderate	(ref)		(ref)	(ref)
	High	−34.2		−60.0, −8.3	0.010
Model 4	Low	−8.1	0.020	−98.4, 82.0	0.859
	Moderate	(ref)		(ref)	(ref)
	High	−33.7		−59.9, −7.5	0.012

CI: confidence interval. BMI: body mass index. HbA1c: glycated haemoglobin. *p*: *p*-value. 25(OH)D cut-offs: <25 nmol/L (low), 25–50 nmol/L (moderate) (ref) and >50 nmol/L (high). Model 1 is the unadjusted model. Model 2 is further adjusted for smoking and alcohol. Model 3 is further adjusted for BMI and physical activity. Model 4 is further adjusted for HbA1c%.

**Table 5:** Association between 25(OH)D cut-offs and telomere length by sex at 18 months.

Sex	Model	25(OH)D	β Coefficient	Adj. R Square	95% CI	*p*
Men (*n* = 304)	Model 1	Low	−3.9	0.041	−147.2, 139.3	0.957
		Moderate	(ref)		(ref)	(ref)
		High	−66.2		−107.3, −25.5	0.002
	Model 2	Low	−11.5	0.036	−156.8, 133.6	0.875
		Moderate	(ref)		(ref)	(ref)
		High	−69.0		−110.4, −27.7	0.001
	Model 3	Low	−16.7	0.037	−167.8, 134	0.827
		Moderate	(ref)		(ref)	(ref)
		High	−70.3		−112.3, −28.3	0.001
	Model 4	Low	−17.6	0.038	−167.6, 132.4	0.817
		Moderate	(ref)		(ref)	(ref)
		High	−65.2		−107.0, −23.3	0.002
Women (*n* = 471)	Model 1	Low	33.3	−0.004	−73.2, 139.8	0.539
		Moderate	(ref)		(ref)	(ref)
		High	−2.3		−34.0, 29.3	0.883
	Model 2	Low	38.2	0.011	−67.4, 143.8	0.477
		Moderate	(ref)		(ref)	(ref)
		High	−2.8		−34.4, 28.6	0.857
	Model 3	Low	21.8	0.004	−90.9, 134.6	0.704
		Moderate	(ref)		(ref)	(ref)
		High	−4.9		−38.2, 28.3	0.770
	Model 4	Low	24.7	0.001	−90.2, 139.6	0.672
		Moderate	(ref)		(ref)	(ref)
		High	−6.5		−40.6, 27.5	0.707

CI: confidence interval. BMI: body mass index. HbA1c: glycated haemoglobin. *p*: *p*-value. 25(OH)D cut-offs: <25 nmol/L, 25–50 nmol/L (moderate) (ref) and >50 nmol/L (high). Model 1 is the unadjusted model. Model 2 is further adjusted for smoking and alcohol. Model 3 is further adjusted for BMI and physical activity. Model 4 is further adjusted for HbA1c%.

**Table 6:** Association between different 25(OH)D cut-offs and Telomere length at 36 months.

Model	25(OH)D	β Coefficient	Adj. R Square	95% CI	*p*
Model 1	Low	−12.6	−0.002	−205.4–180.2	0.898
	Moderate	(ref)		(ref)	(ref)
	High	−28.6		-85.3–28.1	0.322
Model 2	Low	−18.4	−0.007	−213.3–176.4	0.853
	Moderate	(ref)		(ref)	(ref)
	High	−29.6		−86.8–27.6	0.310
Model 3	Low	−5.7	−0.003	−209.5–198.1	0.956
	Moderate	(ref)		(ref)	(ref)
	High	−38.1		−96.5–20.1	0.199
Model 4	Low	16.2	−0.004	−189.8–222.3	0.877
	Moderate	(ref)		(ref)	(ref)
	High	−36.8		−96.3–22.6	0.225

CI: confidence interval. BMI: body mass index. HbA1c: glycated haemoglobin. *p*: *p*-value. 25(OH)D cut-offs: <25 nmol/L (low), 25–50 nmol/L (moderate) (ref) and >50 nmol/L (high). Model 1 is the unadjusted model. Model 2 is further adjusted for smoking and alcohol. Model 3 is further adjusted for BMI and physical activity. Model 4 is further adjusted for HbA1c.

**Table 7:** Association between different 25(OH)D cut-offs and Telomere length by sex at 36 months.

Sex	Model	25(OH)D	B Coefficient	Adj. R Square	95% CI	*p*
Men (*n* = 304)	Model 1	Low	12.7	−0.013	−295.0–320.5	0.935
		Moderate	(ref)		(ref)	(ref)
		High	−1.1		−89.7–87.4	0.980
	Model 2	Low	−2.1	−0.025	−315.1–310.8	0.989
		Moderate	(ref)		(ref)	(ref)
		High	−4.7		−94.5–84.9	0.916
	Model 3	Low	−10.9	−0.018	−33.9–317.1	0.948
		Moderate	(ref)		(ref)	(ref)
		High	−11.9		−103.3–79.4	0.797
	Model 4	Low	−12.1	−0.015	−103.3–82.7	0.827
		Moderate	(ref)		(ref)	(ref)
		High	−10.2		−173.3–89.5	0.530
Women (*n* = 471)	Model 1	Low	−34.0	−0.002	−286.9–218.9	0.791
		Moderate	(ref)		(ref)	(ref)
		High	−45.1		−120.1–29.8	0.237
	Model 2	Low	−33.5	−0.010	−288.1–221.0	0.795
		Moderate	(ref)		(ref)	(ref)
		High	−44.5		−120.2–31.0	0.247
	Model 3	Low	−32.3	−0.009	−300.0–235.4	0.812
		Moderate	(ref)		(ref)	(ref)
		High	−53.3		−131.0–24.2	0.177
	Model 4	Low	−0.8	−0.008	−271.3–269.6	0.995
		Moderate	(ref)		(ref)	(ref)
		High	−50.5		−129.8–28.8	0.211

CI: confidence interval. BMI: body mass index. HbA1c: glycated haemoglobin. *p*: *p*-value. 25(OH)D cut-offs: <25 nmol/L (low), 25–50 nmol/L (moderate) (ref) and >50 nmol/L (high). Model 1 is the unadjusted model. Model 2 is further adjusted for smoking and alcohol. Model 3 is further adjusted for BMI and physical activity. Model 4 is further adjusted for HbA1c.

## Data Availability

Data from the Newcastle 85+ Study is available through a formal application process to the study team. For details please visit https://research.ncl.ac.uk/85plus/datarequests/.

## Supplementary Materials

The following are available online at https://www.mdpi.com/article/10.3390/nu13124341/s1. Supplemental Box S1: List of diseases collected from GP medical records.
