# Supplementary material for: The Association between 25-Hydroxyvitamin D Concentration and Telomere Length in the Very-Old: The Newcastle 85+ Study
Source: Nutrients. 2021 Dec 1;13(12):4341. doi: 10.3390/nu13124341 (PMC8707859; doi:10.3390/nu13124341)
Supplement: Supplementary file 1 [file nutrients-13-04341-s001.zip › nutrients-1413676-supplementary.pdf]

nutrients MDPI

Supplemental Box S1: List of diseases collected from GP medical records

- Hypertension
- Ischaemic heart disease
- Cerebrovascular disease
- Peripheral vascular disease
- Heart failure
- Atrial flutter or fibrillation
- Arthritis (osteoarthritis or cervical or lumbar spondylosis or rheumatoid arthritis or other arthritis or non-specified arthritis)
- Osteoporosis
- Chronic obstructive pulmonary disease or asthma
- Other respiratory disease
- Diabetes
- Hypothyroidism or hyperthyroidism
- Cancer diagnosed within five years (exclusion non-melanoma skin cancer)
- Dementia
- Parkinson’s disease

* Reference [16]
